# Mechanism Underlying Heat Stability of the Rice Endosperm Cytosolic ADP-Glucose Pyrophosphorylase

**DOI:** 10.3389/fpls.2019.00070

**Published:** 2019-02-11

**Authors:** Seon-Kap Hwang, Salvinder Singh, Jitendra Maharana, Samhita Kalita, Aytug Tuncel, Tanmayee Rath, Debashish Panda, Mahendra Kumar Modi, Thomas W. Okita

**Affiliations:** ^1^Institute of Biological Chemistry, Washington State University, Pullman, WA, United States; ^2^Department of Agricultural Biotechnology, Assam Agricultural University, Jorhat, India; ^3^Distributed Information Centre (DIC), Department of Agricultural Biotechnology, Assam Agricultural University, Jorhat, India

**Keywords:** starch synthesis, rice endosperm, AGPase, heat stability, reductive activation, 3-PGA, molecular dynamics simulation

## Abstract

Rice grains accumulate starch as their major storage reserve whose biosynthesis is sensitive to heat. ADP-glucose pyrophosphorylase (AGPase) is among the starch biosynthetic enzymes severely affected by heat stress during seed maturation. To increase the heat tolerance of the rice enzyme, we engineered two dominant AGPase subunits expressed in developing endosperm, the large (L2) and small (S2b) subunits of the cytosol-specific AGPase. Bacterial expression of the rice S2b with the rice L2, potato tuber LS (pLS), or with the mosaic rice-potato large subunits, L2-pLS and pLS-L2, produced heat-sensitive recombinant enzymes, which retained less than 10% of their enzyme activities after 5 min incubation at 55°C. However, assembly of the rice L2 with the potato tuber SS (pSS) showed significantly increased heat stability comparable to the heat-stable potato pLS/pSS. The S2b assembled with the mosaic L2-pLS subunit showed 3-fold higher sensitivity to 3-PGA than L2/S2b, whereas the counterpart mosaic pLS-L2/S2b showed 225-fold lower sensitivity. Introduction of a QTC motif into S2b created an N-terminal disulfide linkage that was cleaved by dithiothreitol reduction. The QTC enzyme showed moderate heat stability but was not as stable as the potato AGPase. While the QTC AGPase exhibited approximately fourfold increase in 3-PGA sensitivity, its substrate affinities were largely unchanged. Random mutagenesis of S2b^QTC^ produced six mutant lines with elevated production of glycogen in bacteria. All six lines contained a L379F substitution, which conferred enhanced glycogen production in bacteria and increased heat stability. Modeled structure of this mutant enzyme revealed that this highly conserved leucine residue is located in the enzyme’s regulatory pocket that provides interaction sites for activators and inhibitors. Our molecular dynamic simulation analysis suggests that introduction of the QTC motif and the L379F mutation improves enzyme heat stability by stabilizing their backbone structures possibly due to the increased number of H-bonds between the small subunits and increased intermolecular interactions between the two SSs and two LSs at elevated temperature.

## Introduction

Starch is a major component in many plant seed endosperms, accounting for 56–74% of the available carbohydrates in the grain of most food crops including cereals (Koehler and Wieser, 2013). ADP-Glucose pyrophosphorylase (AGPase) plays an essential role in glycogen biosynthesis in bacteria (Preiss, 1984, 1996) and is ubiquitous in starch-synthesizing plant tissues (Lee et al., 2007). AGPase catalyzes the initial step in the starch biosynthetic pathway by converting ATP and α-glucose-1-posphate (Glc1P) to ADP-glucose and pyrophosphate (PPi). Evidence from previous studies indicate that AGPase catalyzes a major limiting step in starch synthesis. Transgenic cereals over-expressing modified forms of AGPase showed not only an enhanced rate of starch synthesis but increased final grain yield (Stark et al., 1992; Giroux et al., 1996; Sakulsingharoj et al., 2004; Obana et al., 2006; Gibson et al., 2011). Moreover, null AGPase mutants showed decreased starch content (Johnson et al., 2003) while desensitization of AGPase activity to Pi inhibition led to increased seed yield (Giroux et al., 1996; Smidansky et al., 2003; Cross et al., 2004).

Plant AGPases possess a heterotetrameric structure composed of two small subunits (SS) and two large subunits (LS). Both subunit types evolved from a common progenitor (Georgelis et al., 2007) and each exhibits a defective catalytic and/or regulatory activity when expressed alone in *Escherichia coli* (Iglesias et al., 1993; Burger et al., 2003; Hwang et al., 2008). Plant AGPase activity can be modulated by several mechanisms: allosteric regulation by small effecter molecules, thermal inactivation, and reductive activation. Plant AGPase is activated by 3-phosphoglyceric acid (3-PGA) and inhibited by inorganic phosphate (Pi). The allosteric regulatory properties being a product of synergistic interactions between the large and small subunits (Hwang et al., 2005).

Extreme temperatures are responsible for reduced grain yield and quality worldwide of many cereal crops such as maize (Singletary et al., 1993, 1994), wheat (Asseng et al., 2011), barley (Wallwork et al., 1998b) and rice (Peng et al., 2004; Ahmed et al., 2015). One critical factor influencing yield is starch synthesis, which is highly sensitive to heat stress due to the susceptibility of several biosynthetic enzymes including AGPase in the developing seeds to high temperature (Wallwork et al., 1998a; Ahmed et al., 2015), Since AGPase is a rate-limiting enzyme in starch biosynthesis, the adverse effects of high temperature on the enzyme activity would significantly reduce starch production and, in turn, yield. In contrast to the potato tuber enzyme which is almost fully stable at 60–70°C (Ballicora et al., 1995; Greene and Hannah, 1998; Hwang et al., 2008), AGPases from cereal plants are readily denatured at these elevated temperatures. For example, the maize endosperm AGPase loses 74 ∼ 96% and of its activity when heated at 57 ∼ 60°C for 5 min (Hannah et al., 1980; Greene and Hannah, 1998; Boehlein et al., 2008). Our preliminary analysis showed that the AGPase L2/S2b enzyme, the major seed cytosolic form in rice endosperm, is heat sensitive as it loses nearly all of its catalytic activity when incubated for 5 min at 55°C. Introduction of a heat-stable, phosphate-insensitive maize AGPase mutant into wheat, rice (Smidansky et al., 2003), and maize (Giroux et al., 1996; Smidansky et al., 2002) increased grain yield. Thus, development of heat-stable AGPases from cereal endosperm is a viable approach to increase the potential for better crop yield and quality.

The rice genome contains AGPase genes for four large subunits (L1–L4) and two small subunits (S1 and S2). Interestingly, the S2 gene produces two RNA transcripts, S2a and S2b, via alternative splicing. While S2b is a cytosolic form abundantly expressed in rice endosperm cells (Lee et al., 2007), S2a is a plastidial form expressed in leaves. L3/S2a is believed to be involved in the synthesis of transitory starch in rice leaf tissues while the spatial distribution of L4 is unknown. While the plastidial L1/S1 is predominantly present in amyloplasts at an early stage of grain development, starch biosynthesis is controlled predominantly by the catalytic activity of the cytosolic L2/S2b and its allosteric regulation by metabolic effectors (Tuncel et al., 2014b).

AGPase from potato tuber is naturally thermostable (Boehlein et al., 2008), a property due to the formation of a Cys–Cys disulfide bond between its two SSs (Jin et al., 2005). Comparison of heat-stable and heat-labile AGPases (Ballicora et al., 1999; Linebarger et al., 2005) identified a conserved amino acid motif in the N-terminus of the small subunit of heat-stable enzymes, designated QTCL (Gln-Thr-Cys-Leu), which contains the Cys residue responsible for disulfide bond formation between the pair of SSs. This motif is absent in the heat-labile AGPases of rice and maize endosperms. Insertion of Cys in the N-terminus of the maize AGPase SS enhanced the heat stability of the resulting heterotetrameric enzyme up to 57°C (Linebarger et al., 2005).

Here, we examined the role of the rice AGPase LS on heat stability by using hybrid and mosaic forms of rice AGPase L2 and potato tuber AGPase LS. We also tested the effect of the QTCL motif inserted into S2b (QTC mutation) on heat stability of rice AGPase. In addition, a random mutagenesis on the S2b^QTC^ produced the PQ6 mutant that exhibited further increases in heat stability. These mutants show increased 3-PGA sensitivity but unaltered catalytic activities compared to wild type AGPase both under non-reduced and reduced conditions.

## Materials and Methods

### Construction of Mosaic Proteins and Site-Directed Mutagenesis

In order to construct mosaic L2-pLS, the N-terminal region (Met-1 to Asp-348) of the rice L2 on pAT16 (Tuncel et al., 2014a,b) and the C-terminal region (Ile-294 to Ile-463) of the potato pLS on pSH275A (Hwang et al., 2005, 2008) were amplified by PCR using primers QE34/L2fus_RV-R and Lfus_RV-F/274seq-r (Supplementary Table S1), respectively. The PCR product for the N-terminal domain of L2 was digested with *Nco*I and *Eco*RV and cloned into pGEM58ZNf(-) (Genbank accession no. AF310245) to produce pGEM58_L2-N. Then, the PCR product for the C-terminal domain of pLS was digested with *Eco*RV and *Sac*I and cloned into pGEM_L2-N to produce pSH708, a plasmid DNA expressing a mosaic L2-pLS (Supplementary Figure S1). The mosaic pLS-L2 (pSH709) was constructed in a similar way after PCR amplification using primers QE34/Lfus_RV-R (Met-1 to Asp-293) and L2fus_RV-F/274seq-r (Ile-349 to Ile-518) of pSH275A and pAT16, respectively. The K478N substitution on L2-pLS was performed on pSH708 by using a site-directed mutagenesis method with primers L2pLS_K471N-F/L2pLS_K471N-R. Replacement of Asn38-Lys39-Asn40 (NKN) with Gln-Thr-Cys (QTC) on S2b was performed using Turbo Pfu DNA polymerase with primers S2b-QTC-F and S2b-QTC-R. The modified DNA fragments were then digested with *Nco*I and *Sac*I restriction endonucleases and cloned into protein expression vectors, pSH558 for small subunit DNAs and pSH280 for large subunit DNAs. The plasmids were then transformed into *E. coli* strain EA345 (Hwang et al., 2007; Hwang et al., 2008) to express the wild type and mutant AGPases (Supplementary Figure S1). Glycogen production was determined by exposing bacterial colonies to iodine vapor.

### Random Mutagenesis of Rice AGPase S2b

Random mutagenesis of rice AGPase S2b^QTC^ with hydroxylamine-HCl was performed as described earlier (Hwang et al., 2008). Plasmid DNAs treated with the mutagen were then transformed into *E. coli* EA345 containing AGPase L2 gene for co-expression of the two subunits. Colonies formed on phosphate-enriched Kornberg medium (Hwang et al., 2004) were then stained with iodine vapor to identify mutants with increased AGPase activity and/or heat stability.

### Expression and Purification of Recombinant Potato and Rice AGPases From *E. coli*

*Escherichia coli* EA345 cells (Hwang et al., 2008) expressing recombinant AGPases were grown in modified NZCYM medium (10 g/L Tryptone, 5 g/L yeast extract, 5 g/L NaCl, 1 g/L casamino acids, and 1 g/L MgSO_4_⋅7H_2_O) at 37°C. When the optical density of the culture at 600 nm reached 0.8 ∼ 1.0, the cells were induced with 0.2 mM IPTG and grown at room temperature for another 18 h. The cells were then harvested and lysed by sonication in a buffer containing 25 mM HEPES, pH 8.0, 5% glycerol, 100 μg/mL lysozyme, 100 μM phenylmethylsulfonyl fluoride and 2 μM each of pepstatin A and leupeptin. After clarification by centrifugation the AGPase enzymes were purified as described (Hwang et al., 2008).

### Enzyme Assay and Enzyme Kinetics

Unless otherwise indicated, all AGPase reactions were performed at room temperature and the reaction product (Pi) measured by a modified PAMb method (Hwang et al., 2010; Hwang et al., 2016). Standard reaction mixture (90 μL) in a 96-well plate contained 50 mM Tricine-NaOH, pH 8.0, 5 mM DTT, 10 mM MgCl_2_, 0.15 U inorganic pyrophosphatase, 2 mM ATP, 5 mM 3-PGA, and 1.5 mM Glc1P. Reaction was initiated by adding 10 μL of enzyme solution to the pre-warmed reaction mixture. After 10 min, the reaction was stopped by adding 100 μL of freshly prepared mPAMb solution (1 part of 12% w/v ascorbic acid in 1 M H_2_SO_4_ and 2 parts of 20 mM ammonium molybdate in 1 M H_2_SO_4_). Exactly after 5 min, 100 μL of STOP solution (0.5 M citric acid) was added and absorbance was measured at 650 nm using a SPECTRAmax plus microplate spectrophotometer (Molecular Devices). Potassium phosphate (pH 7.0) was used as the standard. The 4-parameter Hill equation (Hwang et al., 2007) was used to calculate *A*_0.5_, *S*_0.5_, and *V*_max_ values using Kaleidagraph 4.5 (Synergy Software).

### Heat Stability Assay

Wild type and variants of potato and rice AGPases were incubated at 37 and 55°C in 50 mM HEPES-NaOH, pH 7.5, 5 mM MgCl_2_, 0.5 mM EDTA and enzyme activity determined as described earlier. Residual activity (%) is defined as the percentage of enzyme activity after thermal denaturation at 55°C compared to that of the same enzyme kept at 37°C.

### Disulfide Bridge Reduction and Immunoblot Analysis

The wild type rice AGPase (L2/S2b), wild type potato AGPase (pLS/pSS) and mutant AGPases were boiled for 5 min in SDS sample buffer containing 62.5 mM Tris-HCl (pH 6.8), 2% w/w SDS, 7.5% v/v glycerol in the absence or presence of 10 mM DTT. Two μg of protein were resolved on 10% SDS-PAGE and then subjected to immunoblot analysis using a polyclonal antibody raised against the potato AGPase small subunit.

### 3D Modeling of Rice AGPase Subunits

The 3D homology models of rice AGPase were built for the catalytic and β-helix domain regions (including 12 residues from N-terminal loop adjacent to catalytic region) of AGPase L2 (72–518) and S2b (35–479) using MODELLER 9.17 (Webb and Sali, 2014) using the potato AGPase SS structure (PDB ID: 1YP3) (Jin et al., 2005) as a template. The best 3D models were chosen from about 100 generated models based on their lowest discrete optimized protein energy (DOPE) score. We also constructed mutant models for AGPase S2b (S2b^QTC^ and S2b^QTC+L379F^) using Discovery Studio 3.5. The loop remodeling and structural refinements were performed using GalaxyWEB server (Ko et al., 2012) and the model quality and stereo-chemical properties were inferred using PROCHECK (Laskowski et al., 1993), ProSA (Wiederstein and Sippl, 2007), ProQ (Wallner et al., 2003), and Verify3D (Eisenberg et al., 1997) (Supplementary Table S2).

### Modeling of L2/S2b Heterotetrameric Complexes

Three heterotetrameric complexes (L2/S2b^WT^, L2/S2b^QTC^, and L2/S2b^QTC+L379F^) were generated using PyMOL superimposition protocol (Maharana et al., 2017) based on the potato AGPase SS homotetramer (PDB ID: 1YP3) (Jin et al., 2005) and proposed potato heterotetramer structure (Baris et al., 2009) as templates. ATP molecules were docked in the rice AGPase subunits based on the crystal structure of the potato AGPase SS homotetramer (PDB ID: 1YP3).

### Molecular Dynamics Simulation

To examine the structural stability of modeled complexes, molecular dynamics (MD) simulations were performed using GROMACS 5.1 (Pronk et al., 2013) with CHARRM36 force field (Huang and MacKerell, 2013) under periodic boundary conditions. SwissParam (Zoete et al., 2011) was used to generate the topology parameter of the ATP molecule. The modeled heterotetrameric AGPase complexes (L2/S2b^WT^, L2/S2b^QTC^, and L2/S2b^QTC+L379F^) were solvated using TIP3P water models in individual cubic boxes and electro-neutralized by adding physiological strength (150 mM) of Na^+^ and Cl^-^ ions. To avoid steric clashes and high energy interactions, the simulation systems were energy minimized using a steepest-descent algorithm. The long-range electrostatic interactions were treated using particle mesh Ewald (PME) method. The energy minimized systems were equilibrated in two different phases: NVT and NPT for 0.1 and 1 ns, respectively, at 37 and 55°C temperature conditions and the NPT ensemble final production runs were performed at 100 ns timescale.

After obtaining the simulation trajectories (the files containing structural information), we calculated the backbone root mean square deviation (RMSD) using the *gmx rms* program to infer the structural stability of the complex during dynamics. To decipher the nature of intermolecular interaction governed by N terminal loop regions (Arg-72 to Ala-83 in L2:L2′ and Ser-35 to Asp-46 in S2b:S2b′ interface), we calculated the total numbers of intermolecular H-bonds as a function of simulation time using *gmx hbond* and studied the intermolecular interactions. In order to complement the intermolecular H-bonds, we also generated the average contact maps (distance matrices consisting of the smallest distance between residue pairs) between the N-terminal loop regions using *gmx mdmat* tool. PyMOL was used for molecular visualization and interaction analysis and Grace 5.1.2^1^ for 2D graph preparation.

## Results

### N-Terminal Region of Rice AGPase L2 Is Important for 3-PGA Sensitivity

**FIGURE 1:**
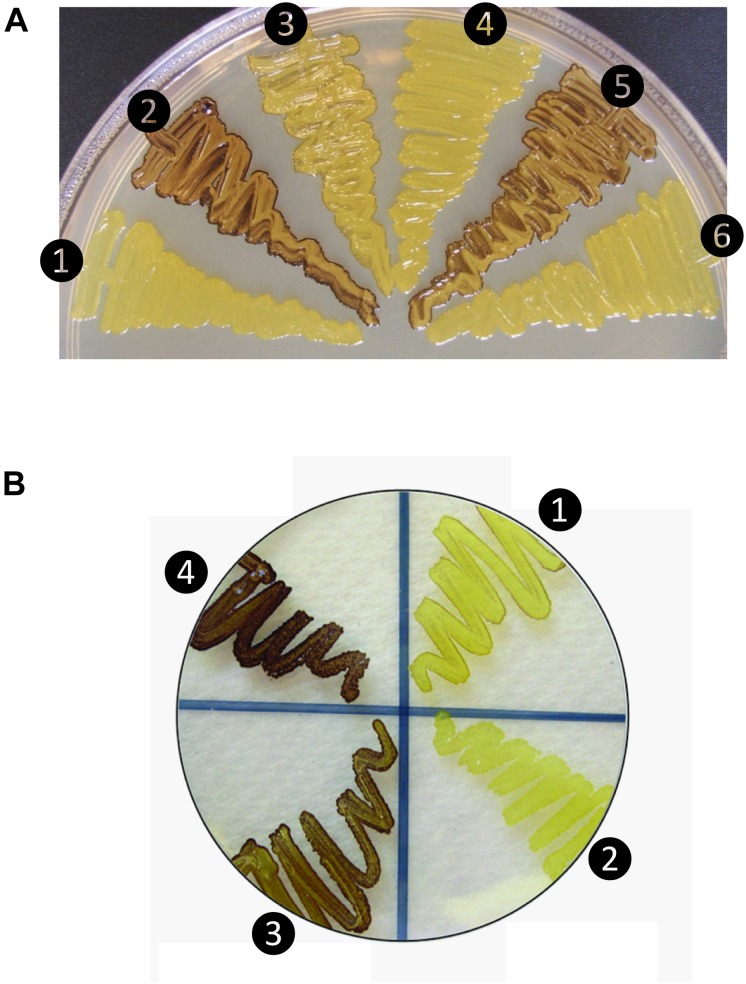
Glycogen production profiles of AGPase variants. **(A)** Rice AGPase S2b subunit were co-expressed in *Escherichia coli* EA345 (Δ*glgC*) with: 1, no LS; 2, rice AGPase L2 (L2); 3, potato tuber LS (pLS); 4, pLS:L2; 5, L2:pLS; and 6, L2:pLS^K471N^. K471N: lysine-471 was substituted with arginine on the β-helix domain of potato LS. Overnight grown cells were exposed to iodine vapor for glycogen staining. Exposure time: 2 min. **(B)** Rice AGPase L2 subunit were co-expressed with: 1, S2b; 2, green fluorescent protein (GFP, negative control); 3, S2b^QTC^ mutant. 4: wild type potato tuber AGPase used as positive control. Exposure time: 30 s. Note the exposure time to iodine vapor was adjusted to emphasize the difference in glycogen production between the potato pLS/pSS and rice L2/S2b.

When co-expressed in *E. coli*, the rice AGPase L2/S2b was capable of producing glycogen (Figure 1A), although at a much lower extent than that evident for the potato pLS/pSS (Figure 1B). Cells expressing the rice S2b with potato tuber LS (pLS) produce low levels of glycogen as viewed by the faint I_2_ staining. Mosaic rice-potato LS enzymes (Supplementary Figure S1) were also tested. These were pLS-L2 where the potato LS N-terminal region was fused to the rice L2 β-helix domain and L2-pLS where the rice L2 N-terminal region was fused to potato LS β-helix domain. The rice S2b co-expressed with a mosaic pLS-L2 produced low levels of glycogen, whereas the counterpart L2-pLS/S2b produced levels of glycogen production comparable to those observed for the wild type rice AGPase. These results indicate that the N-terminal region of L2 is essential for enzyme activity when assembled with S2b. A mutation (K478N) in the C-terminal section of the mosaic large subunit (L2-pLS^K478N^) rendered the resulting L2-pLS^K471N^/S2b enzyme inactive. This K478 residue, located on the C-terminal domain of pLS, corresponds to the a potential effector binding site K404 of the potato SS (Ballicora et al., 1998), is critical for enzyme activity.

When partially purified AGPases were assayed for their sensitivities to 3-PGA in the presence of 5 mM DTT (Table 1), the potato AGPase (pLS/pSS) showed considerably higher sensitivity (*A*_0.5_ = 0.043 mM) compared to the rice L2/S2b (*A*_0.5_ = 0.342 mM) or hybrid pLS/S2b (*A*_0.5_ = 0.711 mM). The hybrid L2/pSS showed more than twofold higher sensitivity (*A*_0.5_ = 0.121 mM) and 1.7-fold higher catalytic activity than the rice wild type L2/S2b. Interestingly, the mosaic L2-pLS/S2b showed increased sensitivity (*A*_0.5_ = 0.121 mM) compared to that of wild type rice enzyme while the counterpart mosaic pLS-L2/S2b showed very poor sensitivity (*A*_0.5_ = 77 mM). These results are consistent with the capacity of these enzymes to produce glycogen in *E. coli* cells (Figure 1A). These results also indicate that the rice S2b enzyme containing the mosaic large subunit having the N-terminal domain of the rice AGPase L2 and the C-terminal domain of the potato AGPase LS (L2-pLS) is significantly more sensitive to 3-PGA activation than the S2b enzyme containing pLS-L2.

**Table 1:** Sensitivity of the AGPase variants to 3-PGA activation.

LS	SS	*A*_0.5_ (mM)	nH	V max (μmol/min/mg)
pLS	pSS	0.043±0.006	0.7±0.02	33.4±1.70
L2	S2b	0.342±0.030	1.2±0.02	6.5±0.37
pLS	S2b	0.711±0.048	0.8±0.04	5.4±0.05
L2	pSS	0.147±0.003	0.6±0.04	11.1±0.04
L2-pLS	S2b	0.121±0.014	0.8±0.06	4.2±0.09
pLS-L2	S2b	77.0±5.8	0.4±0.01	1.7±0.06


*Kinetic parameters were determined at various concentrations of 3-PGA in the presence of saturating concentrations of substrates (2 mM ATP and 1.5 mM Glc 1P) under reduced condition. The A_*0.5*_ value corresponds to the 3-PGA concentration (mM) required for 50% maximal activation. The data are mean values (±SE) obtained from at least two independent experiments with each experiment containing two replicates. nH, Hill coefficient; V_*max*_, maximal specific activity.*

### Rice AGPase Is Heat-Labile and AGPase Small Subunit Is Important for Heat Stability

**FIGURE 2:**
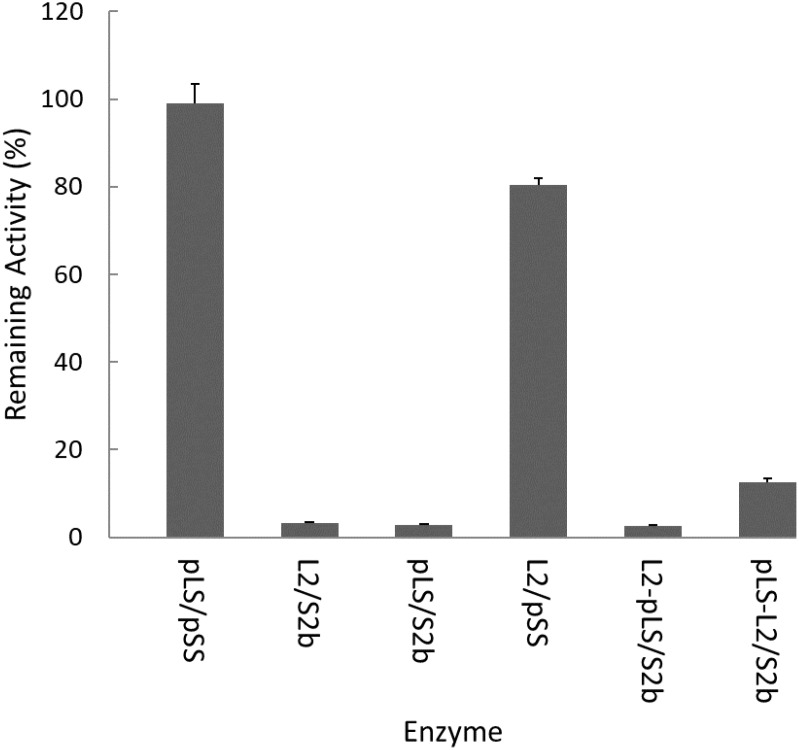
Heat stability of AGPase variants at 55°C. Partially purified AGPase variants were assayed to measure their residual activity, i.e., the percentage of remaining enzyme activity after heat denaturation at 55°C for 5 min compared to that measured after incubation at 37°C for 5 min. The data are mean values (±SE) obtained from at least two independent experiments with two replicates performed at each experiment under standard reaction conditions (see section “Materials and Methods”).

The rice AGPase has been suggested to be a major starch biosynthetic enzyme affected by heat stress (Ahmed et al., 2015). To determine the heat stability of the rice cytosolic AGPase (L2/S2b) we treated the enzyme at 55°C for 5 min and measured its enzyme activity. Purified potato tuber AGPase (pLS/pSS) was used as a positive control. As expected, the potato AGPase was heat stable, whereas the rice AGPase was heat unstable showing less than 2% of residual activity (Figure 2). We also examined the hybrid enzymes, pLS/S2b and L2/pSS, containing reciprocal combinations of potato and rice AGPase subunits to determine which subunit was responsible for heat stability or lability. When the rice L2 was assembled with potato SS (L2/pSS), the resulting enzyme showed about 80% of residual activity, whereas the reciprocal pair, pLS/S2b, showed very little, if any, activity. These results indicate that the potato SS but not the rice S2b possesses elements conferring heat stability. Mosaic proteins of potato and rice AGPase LS were also used to determine if either the N- or C-terminal region of rice L2 or potato LS is important for heat stability. When mosaic proteins (pLS-L2 or L2-pLS) were co-expressed with S2b, both enzymes retained very low enzyme activities after heat treatment although pLS-L2/S2b retained slightly higher activity than L2-pLS/S2b.

### Introduction of a Disulfide Bond Between S2b Subunits Confers Increased Heat Stability

**FIGURE 3:**
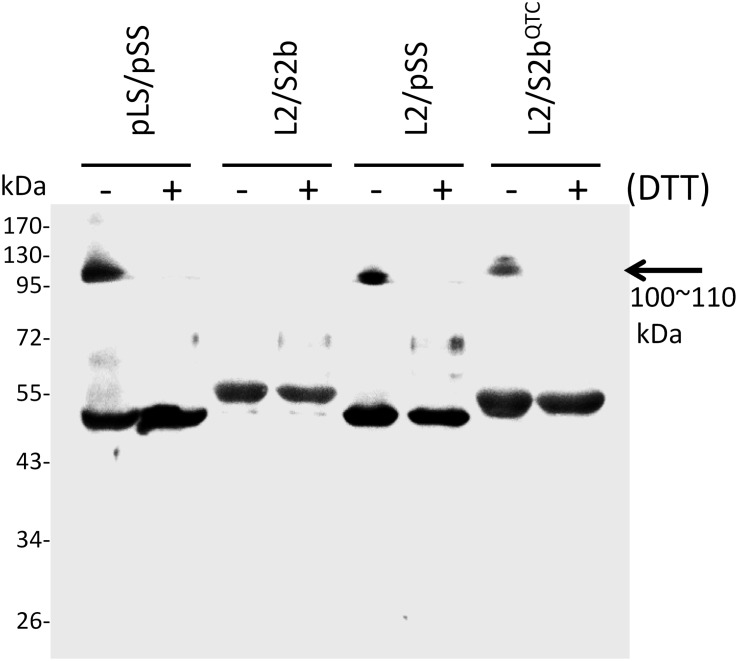
Immunoblot analysis of dimer formation of AGPase variants. 0.2 μg of partially purified enzymes (pLS/pSS, L2/S2b, L2/pSS, and L2/S2b^QTC^) were boiled for 5 min in a SDS sample buffer containing 62.5 mM Tris-HCl (pH 6.8), 2% w/w SDS, 7.5% v/v glycerol in the absence (–) or presence (+) of 10 mM DTT. The samples were resolved by SDS-PAGE and analyzed by immunoblotting using anti-potato tuber AGPase SS. The arrow indicates dimers of potato AGPase SS (pSS) or rice AGPase S2b (S2b^QTC^).

In order to test whether the introduction of disulfide bond between the two S2b subunits also increases the heat stability of the rice AGPase as demonstrated for the maize AGPase (Linebarger et al., 2005), we replaced the N-terminal NKN peptide of S2b with QTC to generate QTCL, a peptide conserved in heat stable AGPases. The resulting S2b^QTC^ was then co-expressed with the L2 subunit in *E. coli*. When cells harboring the QTC mutant (L2/S2b^QTC^) and other variants of AGPase (pLS/pSS, L2/S2b, and L2/pSS) were grown at 37°C and exposed to iodine vapor to measure glycogen production, the potato AGPase as well as the QTC mutant showed significantly higher amounts of glycogen synthesis compared to the wild type rice AGPase (Figure 1B). To determine whether the QTC replacement results in the formation of a Cys–Cys disulfide bond between the two small subunits, the QTC mutant and other AGPase variants were expressed, their proteins purified, and resolved by 10% SDS-polyacrylamide gel electrophoresis (Supplementary Figure S2). When analyzed by immunoblot analysis using the anti-potato SS antibody, the potato protein showed a polypeptide band at 100 kDa in the absence of DTT indicating formation of a dimer between the two pSSs via their N-terminal Cys residues (Figure 3). A SS dimer band was also detected for L2/pSS that retained a substantial level of enzyme activity after heat treatment (Figure 2). This 100 kDa polypeptide band was not detected when these enzymes were incubated with DTT. On the other hand, a 106 kDa dimer band was not evident for the rice enzyme under non-reduced conditions indicating such an inter-subunit disulfide bridge does not exist. A dimer band at 106 kDa was detected for the QTC enzyme (L2/S2b^QTC^) in the absence of DTT but disappeared after incubation with DTT, indicating the QTC replacement created an inter-molecular disulfide bridge between the two S2b^QTC^ subunits. On the other hand, a 106 kDa dimer band was not evident for the rice enzyme under non-reduced conditions indicating such an inter-subunit disulfide bridge does not exist. A dimer band at 106 kDa was detected for the QTC enzyme (L2/S2b^QTC^) in the absence of DTT but disappeared after incubation with DTT, indicating the QTC replacement created an inter-molecular disulfide bridge between the two S2b^QTC^ subunits as demonstrated for the maize enzyme (Linebarger et al., 2005). Although we have not excluded the possibility that this 106 kDa dimer band is a heterodimer of L2- S2b^QTC^, the rice L2 subunit, which itself is redox sensitive, is not capable of assembling with another L2 subunit or with a S2b subunit (Tuncel et al., 2014a). Moreover, our modeled L2/S2b^QTC^ structure shows that the QTC residues of S2b^QTC^ are distal (>28 Å) from any L2 Cys residues. Hence, it unlikely that the 106 kDa band is a L2- S2b^QTC^ dimer, unless the QTC replacement incurred a long-distance conformational change at the interfaces between L2 and S2b, permitting a new disulfide bond(s) between the two subunits.

### QTC and PQ6 Mutants Display Heat Stability

**FIGURE 4:**
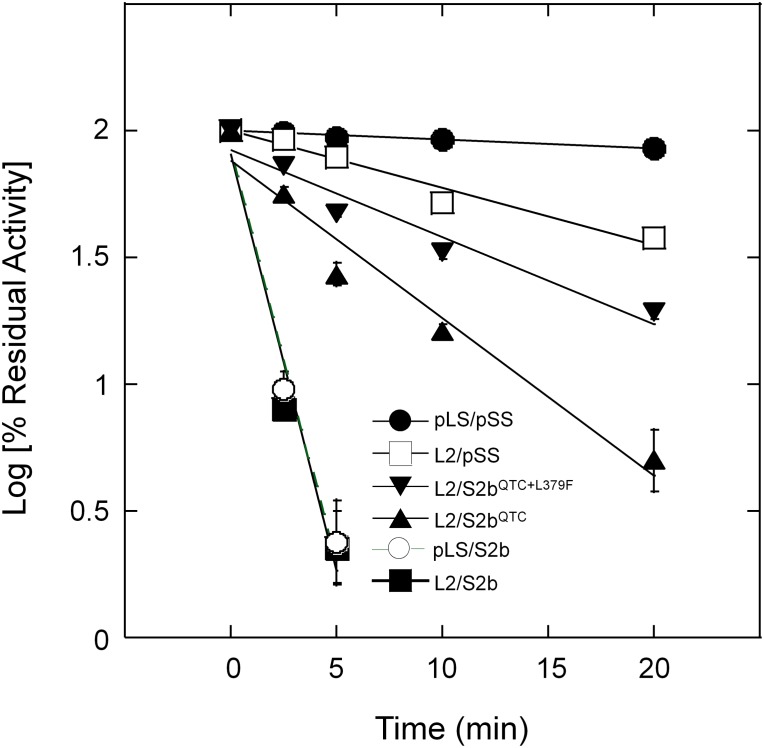
Thermal stability of AGPases. Samples were heat-treated at 55°C for various durations (0 ∼ 20 min) and then immediately placed on ice. Enzyme activity was determined after diluting the protein samples with 50 mM HEPES-NaOH (pH 7) and 5 mM DTT. Enzyme assay was performed under standard condition (see section “Materials and Methods”). Residual activity (%) was calculated with respect to the activity of the control samples not treated with heat. The data points are mean values (±SE) based on the results from three replicate assays. Linear fitting of time versus log (% residual activity) was done with Kaleidagraph 4.5.4 (Synergy Software). No error bars are shown for certain points because they are smaller than the sizes of the symbols.

Purified wild type AGPases (pLS/pSS and L2/S2b) and variants (L2/pSS, pLS/S2b, and L2/S2b^QTC^) were incubated at 55°C up to 20 min and residual activities measured (Figure 4). The wild type rice AGPase was inactivated more than 97% after 5 min incubation, whereas the rice L2 paired with potato SS showed heat stability comparable to the potato enzyme. Conversely, the hybrid pLS/S2b showed very low heat stability much like the rice AGPase. This result indicates that the small subunit of the AGPase enzyme plays a more important role for enzyme’s heat stability than the large subunit. The QTC mutant retained more than 22 and 16% of enzyme activity after 5 min and 10 min incubation at 55°C, respectively, indicating the rice enzyme acquired partial heat stability by the peptide replacement.

To further elevate the enzyme’s activity and/or heat stability of S2b^QTC^ a random mutagenesis approach was employed. Plasmid DNA harboring S2b^QTC^ sequence was treated with the chemical mutagen, hydroxylamine HCl, and transformed into *E. coli* EA345 cells harboring L2. From screening ∼5 × 10^4^ colonies for glycogen accumulation by I_2_ staining, we obtained six colonies displaying elevated staining intensity. When plasmid DNAs of all six mutants were sequenced, all were found to contain a Leu-to-Phe mutation at residue 379 (L379F) in addition to the original QTC replacement. One of them showing the highest level of glycogen production was named PQ6 (S2b^QTC+L379F^). To examine whether this mutation affects heat stability of the enzyme, we incubated the purified PQ6 (L2/S2b^QTC+L379F^) enzyme at 55°C for up to 20 min. The PQ6 mutant retained about 49 and 33% of activity after 5 and 10 min incubation (Figure 4), which is significantly higher than that evident for the QTC mutant.

### Substrate Affinity Was Not Affected by Redox Potential

We were interested on whether the substrate affinity properties of the enzyme were affected by the QTC and L379F mutations and by redox status. When the *S*_0.5_ values for ATP and Glc1P were measured for the enzymes pre-incubated in the absence or presence of 10 mM DTT, both mutants showed similar levels of affinities toward both substrates regardless of the presence or absence of DTT (Table 2). In order to know whether the disulfide bond between two S2b^QTC^ subunits and two S2b^QTC+L379F^ subunits is involved in redox regulation of catalytic turnover rate, we also measured the enzyme’s specific activities of wild type L2/S2b and the mutants. In all cases, the catalytic rates were not affected by the redox status of the enzymes.

**Table 2:** Substrate affinities and reductive activation of AGPases.

Heterotetramer		10 mM DTT	*S*_0.5_ (mM)				*V*_max_ (μmol/min/mg)
			
LS	SS		ATP	nH	Glc1P	nH	
L2	S2b	-	0.170 ± 0.010	1.6 ± 0.11	0.129 ± 0.004	1.1 ± 0.19	7.3 ± 0.25
		+	0.204 ± 0.006	1.5 ± 0.04	0.087 ± 0.001	0.9 ± 0.12	5.7 ± 0.14
L2	S2b^QTC^	-	0.181 ± 0.004	1.4 ± 0.02	0.215 ± 0.01	1.4 ± 0.13	7.5 ± 0.19
		+	0.221 ± 0.007	1.4 ± 0.03	0.217 ± 0.009	1.6 ± 0.09	8.0 ± 0.17
L2	S2b^QTC+L379F^	-	0.253 ± 0.011	1.3 ± 0.04	0.333 ± 0.002	1.5 ± 0.07	6.5 ± 0.13
		+	0.260 ± 0.010	1.4 ± 0.01	0.326 ± 0.038	1.8 ± 0.11	7.1 ± 0.26


*Kinetic parameters, S_*0.5*_ values for ATP and Glc1P and V_*max*_, were determined in the absence (-) or presence (+) of 10 mM DTT. For enzyme assays under reduced condition, the enzymes were pre-incubated with 10 mM DTT for 5 min at room temperature. The S_*0.5*_ value corresponds to the substrate concentration (mM) required for 50% maximal activity. The data are mean values (±SE) obtained from at least two independent experiments with two replicates performed at each experiment. nH, Hill coefficient; V_*max*_, maximal specific activity.*

### 3-PGA Sensitivity Was Increased by the QTC and L379F Mutations

Regulatory properties of the purified enzymes were assayed after the enzymes were pre-incubated in the absence or presence of 10 mM DTT. Overall, incubation of the enzyme with 10 mM DTT significantly increased the enzyme’s affinity to 3-PGA regardless of the source or combinations of AGPase subunits (Table 3). The potato enzyme displays increased sensitivity to 3-PGA after incubation with the reductant, consistent with the previous report (Ballicora et al., 2000). When activities of the non-reduced enzymes were measured, the rice AGPase showed very poor response to 3-PGA (*A*_0.5_ = 3 mM). However, the *A*_0.5_ value of the QTC mutant (*A*_0.5_ = 0.787 mM) was about fourfold lower than that of wild type rice AGPase while that of the PQ6 mutant was (*A*_0.5_ = 0.34 mM) was ninefold lower. Hence, under non-reduced conditions, the QTC mutation increased the rice enzyme’s sensitivity to 3-PGA with a further enhancement by the introduction of the L379F mutation. It should be noted that 3-PGA sensitivity of PQ6 under non-reduced condition is similar to that of wild type rice AGPase under reduced conditions. The QTC and PQ6 mutants showed similar levels of sensitivity under reduced conditions, but they were still more than threefold higher than that of reduced wild type rice AGPase. It is interesting to note that the rice L2 with potato SS showed 3-PGA activation profile similar to that of the PQ6 mutant. The specific activities of the QTC and PQ6 mutants were roughly similar to those of the wild type regardless of their redox statuses at saturated concentrations of 3-PGA and substrates, indicating that the QTC replacement and an additional mutation L379F do not affect catalytic turnover properties of the rice AGPase. These results suggest that the stronger iodine staining exhibited by the QTC and PQ6 expressing bacterial strains were not attributable to changes in the catalytic activity of the mutant enzymes but, instead, to the enzyme’s increased responsiveness to activators and possibly enhanced heat stability.

**Table 3:** 3-PGA sensitivities and reductive activation of AGPase variants.

LS	SS	10 mM DTT	*A*_0.5_ (mM)	Fold Increase^a^	nH	*V*_max_ (μmol/min/mg)
pLS	pSS	-	0.102 ± 0.007		1.1 ± 0.06	31.8 ± 0.80
		+	0.043 ± 0.006	2.4	0.6 ± 0.06	34.0 ± 1.55
L2	S2b	-	2.995 ± 0.418		1.0 ± 0.03	7.2 ± 0.68
		+	0.342 ± 0.030	8.8	1.4 ± 0.12	7.3 ±0.25
L2	pSS	-	0.322 ± 0.005		1.8 ± 0.04	9.8 ± 0.18
		+	0.147 ± 0.003	2.2	0.7 ± 0.00	10.8 ± 0.01
L2	S2b^QTC^	-	0.787 ± 0.075		2.1 ± 0.02	5.0 ± 0.10
		+	0.095 ± 0.002	8.3	1.3 ± 0.02	6.3 ± 0.09
L2	S2b^QTC+L379F^	-	0.340 ± 0.014		1.9 ± 0.01	6.3 ± 0.17
		+	0.106 ± 0.000	3.2	1.5 ± 0.06	6.6 ± 0.15


*^*a*^Fold Increase: A_*0.5*_ value without pre-incubation with DTT/A_*0.5*_ value after pre-incubation with DTT. Kinetic parameters were determined in the absence (-) or presence (+) of 10 mM DTT at saturating concentrations of substrates (2 mM ATP and 1.5 mM Glc 1P). For enzyme assays under reduced conditions, the enzymes were pre-incubated with 10 mM DTT for 5 min at room temperature. The A_*0.5*_ value corresponds to the 3-PGA concentration (mM) required for 50% maximal activation. The data are mean values (±SE) obtained from at least two independent experiments with two replicates performed at each experiment. nH, Hill coefficient; V_*max*_, maximal specific activity.*

### Modeling of Heterotetrameric AGPase Complexes Shows a Disulfide Bridge Formation Between S2b Mutants

**FIGURE 5:**
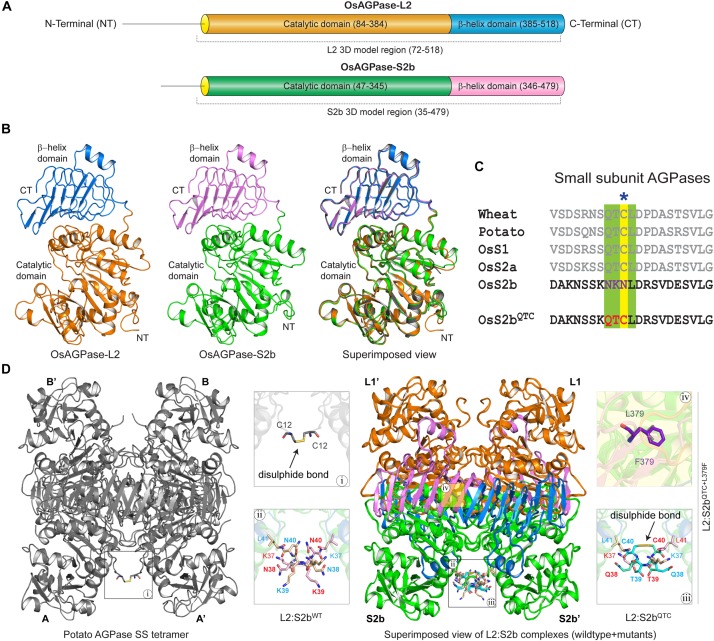
**(A)** Schematic overview of functional domain regions of rice AGPase subunits. The domain regions and connecting linker of both the AGPases are colored (AGPase-L2: orange, catalytic domain; blue, β-helix domain; OsAGPase-S2b: green, catalytic domain; blue, β-helix domain). **(B)** 3D models of OsAGPase-L2 and S2b and their superimposed view with potato SS structure. **(C)** QTCL motifs and inserted QTC in OsS2b shown in multiple sequence alignment of AGPase SSs. **(D)** Overview of potato SS tetramer and modeled L2:S2b and L2:S2b^QTC^ heterotetrameric complexes. Highlighted in magnifying boxes are: (i) Cys–Cys disulfide bond in potato SS; (ii) Asn-Lys-Asn peptide at the N-terminus of rice AGPase; (iii), Cys–Cys disulfide bond between S2b^QTC^; (iv) L379F mutation on S2b^QTC+L379F^.

The domain analysis of the rice AGPase L2 and S2b showed the N-terminal loop peptide regions, Met-1 to Ala-83 for L2 and Met-1 to Asp-46 for S2b, followed by structurally folded regions consisting of the catalytic and β-helix domains (Figure 5A). The initial N-terminal loop peptide regions (Met-1 to Leu-71 for L2 and Met-1 to Asn-34 for S2b) were removed prior to the 3D model building of each subunit. As presented in Figure 5B, the predicted 3D models of AGPase L2 and S2b were found to be very similar to the crystal structure of the potato AGPase SS (1YP3). The qualities, spatial arrangements, and stereochemical parameters of the 3D models were found to be very satisfactory (Supplementary Table S2 and Supplementary Figure S3), indicating that the models were suitable for further structural study.

The S2b isoform of rice AGPase lacks the QTCL motif (Figure 5C) that plays a crucial role in heat stability of the potato enzyme (Ballicora et al., 1999). To understand the impact of QTCL motif in rice S2b, we inserted the ‘QTC’ in place of ‘NKN’ (38–40 aa) in the 3D model of S2b (S2b^QTC^). In addition, as per our experimental findings from the PQ6 mutant, we substituted Leu with Phe at 379th position and generated the S2b^QTC+L379F^ 3D model. Based on the potato AGPase SS homotetramer crystal structure, three heterotetrameric rice AGPase complexes (L2:S2b^WT^, L2:S2b^QTC^, and L2:S2b^QTC+L379F^) (Figure 5D) were generated and used for dynamic study.

### Thermodynamic Stability of L2/S2b^QTC^ Heterotetramer

**FIGURE 6:**
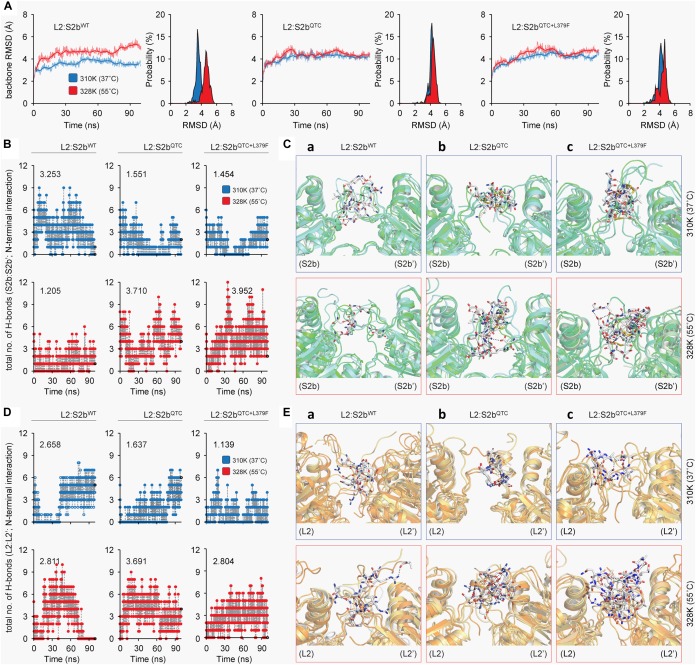
Molecular dynamics analysis of L2:S2b complexes at two different temperature conditions (37 and 55°C). **(A)** Backbone RMSD of L2:S2b complexes (wild type, QTC and PQ6 mutants). **(B)** Intermolecular H-bonds formed in S2b:S2b′ interface (blue, 37°C; red, 55°C). **(C)** Superimposed view of intermolecular interaction(s) at 25 ns between S2b:S2b′ interface (upper panel, 37°C; lower panel, 55°C). **(D)** Intermolecular H-bonds formed at the L2:L2′ interface (blue, 37°C; red, 55°C). **(E)** Superimposed view of intermolecular interaction(s) at 25 ns between L2:L2′ interface (upper panel, 37°C; lower panel, 55°C). The S2b and L2 subunits’ N-terminal loop regions are displayed in light-green and light-orange cartoons, respectively, and the interacting residues are presented in white stick models. The black-dotted lines represent the intermolecular polar contacts between two S2b subunits and between two L2 subunits.

To determine the underlying basis for the higher heat stability displayed by the S2b mutant enzymes, we calculated the backbone root mean square deviations (RMSD) of the three complexes as a function of simulation time. The thermodynamic stability of the wild type complex (L2/S2b^WT^) was affected by higher temperature, whereas it was fairly stable in the mutant complexes (L2/S2b^QTC^ and L2/S2b^QTC+L379F^) (Figure 6A). A stable and lower deviation of ∼3.6 Å was observed for L2/S2b^WT^ complex at 37°C in comparison to almost ∼4.8 Å deviation at 55°C, which tended to increase after 75 ns. However, the mutant complexes showed stable and similar patterns of RMSD at both temperatures. Next, we calculated the total numbers of H-bonds as a function of simulation time and performed the interaction analysis to understand the nature of interactions between the N-terminal loops (S2b:S2b′ and L2:L2′). The total number of H-bond (3.253) at the N-terminal interface of wild type S2b subunits at 37°C drastically dropped to 1.205 when the temperature was increased to 55°C (Figure 6B). However, in the case of the QTC (L2/S2b^QTC^) and PQ6 (L2/S2b^QTC+L379F^) enzymes, while the total numbers of H-bonds at 37°C (1.551 and 1.454, respectively) were less than that of the wild type complex at 37°C, the numbers of H-bonds were significantly increased in the mutant complexes at 55°C (3.710 and 3.952, respectively) (Figure 6B). It was also observed that the PQ6 complex showed a more stable pattern of H-bonding (higher number of H-bonds) than the QTC complex, which might explain why the PQ6 mutant is more heat stable than the QTC mutant. Additionally, as shown in Figure 6C-a, the intermolecular interactions at the S2b:S2b’ loop interface at 55°C were comparatively less than those at 37°C in the wild type L2:S2b complex. By contrast, the intermolecular interactions of the loop interface were more frequent in the mutant enzymes at higher temperature (55°C) (Figure 6C-b,c).

A similar pattern was evident at the L2–L2′ interface. Although the number of H-bond (2.811) was slightly higher at 55°C (2.811) than at 37°C (2.658) for the wild type enzyme, H-bonding ability was totally lost after 80 ns of MD simulation at the elevated temperature (Figure 6D). By contrast, the mutant complexes (L2/S2b^QTC^ and L2:S2b^QTC+L379F^) exhibited stable H-bonding patterns even after 80 ns and the total number of hydrogen bonds at the L2:L2′ interface increased more than twofold at 55°C (Figure 6D). While the intermolecular interactions at L2:L2′ interface of the L2/S2b^WT^ complex was relatively conserved between 37 and 55°C (Figure 6E-a), the N-terminal loop interfaces (L2:L2′) of the mutant complexes formed significantly more intermolecular interactions at the elevated temperature (Figure 6E-b,c). Thus, the insertion of ‘QTC’ in place of ‘NKN’ in the rice S2b subunit increases the stability of the enzyme complex at higher temperature, which was further enhanced by the Leu to Phe at the 379th position.

**FIGURE 7:**
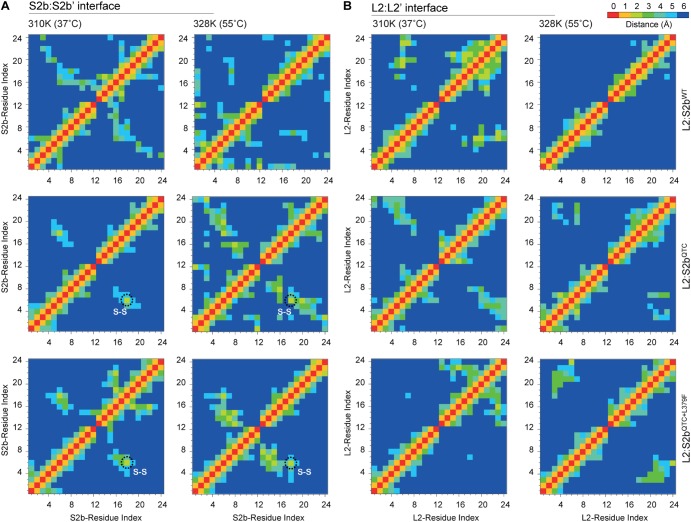
The cross correlation matrix representing the residue pair contact maps between N-terminal loops of **(A)** S2b:S2b′ and **(B)** L2:L2′ interfaces of L2/S2b^WT^, L2/S2b^QTC^, and L2/S2b^QTC+L379F^ complexes at 37 and 55°C. The black dotted circle indicates the Cys–Cys disulfide bonds.

To further analyze the H-bonds and intermolecular interactions, we calculated the smallest distance between the residue pairs of the S2b:S2b′ and L2:L2′ interfaces (Figure 7). In the S2b:S2b′ interface of the L2:S2b^WT^ complex, the calculated distance matrix revealed slightly lower number of residue-residue contacts at 55°C than that at 37°C. In contrast, relatively higher numbers of contacts at 55°C than those at 37°C were evident for the mutant complexes (L2/S2b^QTC^ and L2:S2b^QTC+L379F^) (Figure 7A). Disulfide bridges were readily observed in the mutant complexes at both temperatures. The L2:L2′ interface exhibited a clear difference with respect to the residue interaction (Figure 7B). While dense contacts were observed in the L2:S2b^WT^ complex at 37°C, conserved contacts almost completely disappeared at 55°C. However, considerable numbers of contacts were observed at both temperatures in the mutant complexes. These results are consistent with those from the H-bonding and intermolecular interaction analyses (Figure 6).

## Discussion

Starch production in cereal plants relies heavily on the function of AGPase that catalyzes a rate-limiting step for starch synthesis. Thus, an alteration in the enzymatic properties of this enzyme can profoundly affect starch content and quality (Stark et al., 1992; Giroux and Hannah, 1994; Giroux et al., 1996; Smidansky et al., 2003; Tuncel et al., 2014b). Since yield losses of cereal crops are closely associated with temperature stress (Singletary et al., 1993, 1994), a superior AGPase with increased heat stability and regulatory properties would be beneficial to cereal crop production.

In this study, we determined whether the large subunit of AGPase plays an important role for enzyme’s heat stability. In contrast to the potato tuber AGPase that retained more than 95% activity after heat treatment at 55°C for 5 min, the rice enzyme lost more than 97% activity under the same conditions (Figure 2), indicating that the cytosolic AGPase from rice endosperm is heat labile. When the subunits of potato and rice AGPases were shuffled for co-expression, pLS/S2b showed very low heat stability whereas L2/pSS retained about 80% of activity after heat treatment. These results indicate that the SS is responsible for enzyme’s heat stability.

We also tested whether the N-terminal region (catalytic domain) or C-terminal region (β-sheet domain) of LS is involved in heat stability. Mosaic pLS-L2 and L2-pLS were constructed where the potato LS catalytic domain was fused to the rice L2 β-helix domain and the rice L2 catalytic domain fused to the potato LS β-helix domain, respectively. When co-expressed with S2b, the hybrid enzymes did not show significantly improved heat stability, although the mosaic L2-PLS/S2b enzyme showed considerable catalytic activity. This result indicates that the LS of AGPase is not responsible for heat stability of AGPase. Collectively, our results suggest that heat stability of AGPase is provided by the SS and that the potato SS has intrinsic features contributing to heat stability.

Heat stability on the maize endosperm AGPase was accomplished by placement of the ‘QTCL motif’ into a mosaic MPss containing amino acids 1–199 from the maize endosperm SS and amino acids 200–475 from the potato tuber AGPase SS (Boehlein et al., 2005). Although the substitution of maize SS with MPss increases the heat stability of the resulting heterotetrameric enzyme by fourfold, placement of QTCL into MPss increases heat stability over 300-fold. Previous work with the potato tuber AGPase (Fu et al., 1998) identified this Cys as being important for enzyme stability. This Cys residue is also involved in the reductive activation of the potato AGPase. However, the rice AGPase S2b lacks a Cys residue near the N-terminus and, hence, no disulfide bond is formed between the two S2b subunits (Figure 3). Thus, in order to test whether the rice AGPase could acquire a considerable level of heat stability by introducing a disulfide bond into its SS, we replaced NKN at the N-terminus of S2b with QTC to generate S2b^QTC^. Immunoblot analysis of the purified L2/S2b^QTC^ under non-denaturing condition using anti-potato SS antibody showed a 106-kDa band, indicating formation of dimeric S2b^QTC^.

L2/S2b^QTC^ showed enhanced heat stability compared to L2/S2b and pLS/S2b, which exhibited very poor heat stability (Figure 4). However, the gain in heat stability by L2/S2b^QTC^ was still intermediate to those displayed by the hybrid L2/pSS or potato pLS/pSS. Thus, in an effort to further increase the heat stability of the enzyme, we used a random mutagenesis method to introduce additional mutations(s) into S2b^QTC^ and then identified bacterial colonies exhibiting increased level of glycogen staining. When we sequenced plasmid DNAs from six colonies that showed increased glycogen production, all contained a L379F mutation. Leu-379 is the homologous residue of Leu-351 in the potato AGPase SS and is flanked by conserved amino acids that constitute a β-helix structure. This residue is located at the regulatory pocket where activator and inhibitor molecules bind. The mutant rice PQ6 enzyme, L2/S2b^QTC+L379F^, showed improved heat stability compared to the QTC mutant (Figure 4). These results indicate that QTC replacement and L379F on S2b play a significant role in increasing enzyme’s heat stability. Since the PQ6 enzyme is still not as heat-stable as L2/pSS or pLS/pSS, the possibility remains that replacement of other residues or structural elements, yet-to-be identified, will further improve heat stability.

Expression of the QTC and PQ6 enzymes enhanced glycogen production in bacteria. Thus, we examined whether the catalytic properties of the mutant enzymes were increased. Substrate affinities and specific activities of these enzymes were largely unchanged by the mutations (Table 2), suggesting that the increased glycogen production is not linked to the enzyme’s catalytic properties. Glycogen production was also elevated in bacterial cells expressing the maize QTCL-AGPase (Linebarger et al., 2005). Similar to that seen for the rice QTC mutant, the substrate affinity properties of the maize QTCL enzyme were not altered. The maize QTCL enzyme did display a twofold increase in catalytic activity, indicating that the effects of the QTC replacement on the catalytic properties of the maize and rice enzymes were non-identical.

The catalytic activity of the potato enzyme is regulated by the redox status where a 2.4-fold increase in 3-PGA sensitivity is evident under reducing conditions (Table 3). The hybrid L2/pSS also showed higher 3-PGA sensitivity in the presence of DTT. Interestingly, the catalytic activity of the wild type rice AGPase, which does not form a disulfide bond between its S2b subunits, is still highly redox regulated, consistent with our previous result (Tuncel et al., 2014a). Pre-incubation of the rice enzyme with 10 mM DTT increased 3-PGA sensitivity about 8.8-fold. Both the QTC and PQ6 mutants were also redox-regulated exhibiting increases in 3-PGA sensitivity of 8.3- and 3.2-fold, respectively. The gain in regulatory properties of the QTC and PQ6 enzymes were readily noticeable when their regulatory properties were compared to wild type enzyme. Under non-reduced conditions, the QTC and PQ6 mutants showed 3.8- and 8.8-fold higher sensitivity, respectively, to 3-PGA than the wild type rice AGPase. Under reduced conditions, the QTC and PQ6 mutants have 3.6- and 3.2-fold higher sensitivities, respectively, than that observed under non-reduced conditions. Since the specific activities of the wild type, QTC mutant, and PQ6 mutant were similar, it is likely that the enhanced 3-PGA sensitivity exhibited by the QTC and PQ6 enzymes is the major factor contributing to increased glycogen production in *E. coli*. We cannot rule out a possibility, however, that the enhanced heat stability of the mutants also played a role for the increased glycogen production.

Molecular insights on the interactions between the potato AGPase subunits have been obtained by using structural bioinformatics approaches (Tuncel et al., 2008; Baris et al., 2009). In order to understand the effect of high temperature on the enzymatic properties of L2/S2b^WT^, L2/S2b^QTC^, and L2/S2b^QTC+L379F^ complexes, *in silico* analysis was employed using temperature-dependent MD simulations of their respective modeled heterotetramers (Figure 5) at two different temperatures (37 and 55°C). Our results reveal that heat lability of L2/S2b^WT^ complex might be due to a backbone instability at 55°C as viewed by the significant changes in RMSD. By contrast, L2:S2b^QTC^ and L2/S2b^QTC+L379F^ complexes showed very little changes in RMSD (Figure 6A), indicating the two mutant complexes have stable backbones at elevated temperature. The number of H-bonds and residue pair contacts at the N-terminal interfaces of S2b:S2b and L2:L2 also increased for the mutant enzymes as the temperature increased (Figure 6B,D, 7), which would further stabilize overall structures of the enzymes at elevated temperatures. Past studies (Tuncel et al., 2008; Baris et al., 2009) have suggested that, in addition to the ‘catalytic’ and ‘β-helix’ domain interactions, the N-terminal loops (initial peptide regions) are also involved in intermolecular subunit interaction. The mutants, L2/S2b^QTC^ and L2/S2b^QTC+L379F^, have more interactions between the N-terminal loops of their small subunits (S2b:S2b′ interface) at elevated temperature, whereas the number of interactions in the wild type L2/S2b^WT^ decreases (Figure 6C). Similarly, the mutant AGPases also have more interactions between the N-terminal loops of their large subunits (L2:L2′ interface) at the elevated temperature (Figure 6E). Collectively, our *in silico* analysis of L2/S2b^QTC^ and L2/S2b^QTC+L379F^ indicate that the gain in heat stability is due to the stabilization of the backbone structures (Figure 6A), the increased number of H-bonds between the small subunits (Figure 6B) and large subunits (Figure 6D), and the increased intermolecular interactions between the two SSs and two LSs at elevated temperature (Figure 6C,E).

It should be emphasized that the regulatory properties of the rice L2/Sb2, which lacks a Cys–Cys bridge between its small subunit (S2b) and is heat labile, is still highly modulated by redox, suggesting rice AGPase has a different redox regulation mechanism from other plastidial AGPases including the potato tube AGPase. Interestingly, creation of an intermolecular disulfide bridge between S2b subunits increased the enzyme’s 3-PGA sensitivity by 3.6- to 3.8-fold regardless of its redox status, which is more dramatic than that shown by maize endosperm AGPase with the same QTC replacement (1.5-fold) (Linebarger et al., 2005). Since both wild type and mutant forms of rice AGPase equally show a high degree of redox regulation, we conclude that the disulfide bridge created at the N-terminus of S2b is not primarily involved in redox regulation. It is apparent, however, that the disulfide bridge increases the enzyme’s sensitivity to 3-PGA activation although the underlying mechanism for this enhanced activation is unknown. The L379F mutation further increases the 3-PGA sensitivity of the rice enzyme likely due to its modifying the effector binding site in S2b subunit.

## Conclusion

Cytosolic AGPase from rice endosperm is heat-labile and redox regulated. Introduction of the QTCL motif to create a disulfide bridge between the N-termini of its small subunits had very little effects on the catalytic properties and redox regulation of the enzyme but enhanced its heat stability and regulatory properties. An additional mutation, L379F, on the C-terminal β-helix domain further enhanced heat stability and sensitivity to 3-PGA, a metabolic activator. Dynamic simulation analysis revealed that the increased heat stability exhibited by the mutant complexes is attributed to an increase in the numbers of H-bonds and intermolecular interactions at the N-terminal interfaces of the enzymes’ subunits. The rice AGPase mutants obtained in this study have valuable traits that could be applied to improve starch production in cereal plants especially under heat stress conditions.

## Author Contributions

S-KH and SS conducted most of the experiments and analyzed the results. JM performed the dynamic simulation analysis. SK and AT conducted some of the protein purification and enzyme assays. SS, S-KH, TR, DP, MM, and TO conceived the idea for the project. SS and JM drafted the manuscript. S-KH and TO finalized the manuscript.

## Conflict of Interest Statement

The authors declare that the research was conducted in the absence of any commercial or financial relationships that could be construed as a potential conflict of interest.

## Footnotes

1 http://plasma-gate.weizmann.ac.il/Grace/

## Abbreviations

3-PGA: 3-phosphoglycerate
AGPase: ADP-glucose phosphorylase
DTT: dithiothreitol
Glc1P: glucose 1-phosphate
SDS: sodium dodecyl sulfate
