# Supplementary material for: Mechanism Underlying Heat Stability of the Rice Endosperm Cytosolic ADP-Glucose Pyrophosphorylase
Source: Front Plant Sci. 2019 Feb 11;10:70. doi: 10.3389/fpls.2019.00070 (PMC6378277; doi:10.3389/fpls.2019.00070)
Supplement: Supplementary file 1 [file Data_Sheet_1.pdf]

Supplementary Material

Table S1. Oligonucleotides used in this study.

Name	Sequence	Note
L2fus_RV-F	CACAGAATATTGGGAG<u>GATATC</u>GGAACAATCAAATCATT	Amplification of C-terminal domain of rice AGPase-L2
L2fus_RV-R	AATGATTTGATTGTTCC<u>GATATC</u>CTCCCAATATTCTGTG	Amplification of N-terminal domain of rice AGPase-L2
Lfus_RV-F	CAAAGACTATTGGGAA<u>GATATC</u>GGAACAATTAAATCGTT	Amplification of C-terminal domain of potato AGPase LS
Lfus_RV-R	AACGATTTAATTGTTCC<u>GATATC</u>TTCCCAATAGTCTTTG	Amplification of N-terminal domain of potato AGPase LS
L2pLS_K471N-F	AGGAAATGTATCATTGACAATAACGCAAAGATAGGAAAG	Substitution of Lys-471 with Asn
L2pLS_K471N-R	CTTTCCTATCTTTGCGTTATTGTCAATGATACATTTCCT	Substitution of Lys-471 with Asn
S2b-QTC-F	AACTCGTCCAAGAACAAATGCCTTGACCGCAGTGTCGAT	Substitution of Asn-Lys-Asn witg Gln-Thr-Cys
S2b-QTC-R	ATCGACACTGCGGTCAAGGCATTTGTTCTTGGACGAGTT	Substitution of Asn-Lys-Asn witg Gln-Thr-Cys
QE34	CGGATAACAATTTCACACAG	Amplification of N-terminal domain of rice AGPase L2 and potato AGPase LS
274seq-r	ATCCAGATGGAGTTCTGAGGTCAT	Amplification of C-terminal domain of rice AGPase L2 and potato AGPase LS

Table S2. Structure validation report of AGPase subunit 3D models.

Servers	Scores	AGPase L2	AGPase S2b
PROCHECK	Most favored (%)	91.3	90.7
	Additional allowed (%)	8.4	8
	Generously allowed (%)	0	0.8
	Disallowed (%)	0.3	0.5
ProSA	Z-Score	-9.8	-9.32
ProQ	LGscore	7.511	7.79
	MaxSub	0.657	0.688
Verify 3D	1D-2D profile >0.2 (%)	99.11	99.55

FIGURES S1, S2, and S3

L2 (rice AGPase large subunit)

MQFMMPLDTNACAQPMRRAGEGAGTERLMERLNIGGMTQEKALRKRCFGDGVTGTARCVFTS
DADRDTPHLRTQSSRKNYADASHVSAVILGGGTGVQLFPLTSTRATPAVPVGGCYRLIDIPMSNC
FNSGINKIFVMTQFNSASLNRHIHHTYLGGGINFTDGSVQVLAATQMPDEPAGWFQGTADAIRKF
MWILEDHYNQNNIEHVVILCGDQLYRMNYMELVQKHVDDNADITISCAPIDGSRASDYGLVKF
DDSGRVIQFLEKPEGADLESMKVDTSFLSYAIDDKQKYPYIASMGIYVLKKDVLLDILKSKYAHL
QDFGSEILPRAVLEHNVKACVFTEYWEDIGTIKSFFDANLALTEQPPKFEFYDPKTPFFTSPRYLPP
ARLEKCKIKDAIISDGCSFSECTIEHSVIGISSRVSIGCELKDTMMMGADQYETEEETSKLLFEGKV
PIGIGENTKIRNCIIDMNARIGRNVIIANTQGVQESDHPEEGYYIRSGIVVILKNATIKDGTVI

S2b (rice AGPase small subunit)

MNVLASKIFPSRSNVASEQQQSKREKATIDDAKNSSKNKNLDRSVDESVLGIILGGGAGTRLYPL
TKKRAKPAVPLGANYRLIDIPVSNCLNSNISKIYVLTQFNSASLNRHLSRAYGNNIGGYKNEGFVE
VLAAQQSPDNPNWFQGTADAVRQYLWLFEEHNVMEFLILAGDHLYRMDYEKFIQAHRETDSDI
TVAALPMDEKRATAFGLMKIDEEGRIVEFAEKPKGEQLKAMMVDTTILGLDDVRAKEMPYIAS
MGIYVISKNVMLQLLREQFPGANDFGSEVIPGATNIGMRVQAYLYDGYWEDIGTIEAFYNANLGI
TKKPVPDFSFYDRSAPIYTQPRHLPPSKVLDADVTDSVIGEGCVIKNCKIHHSVVGLRSCISEGAII
EDSLLMGADYYETEADKKLLGEKGGIPIGIGKNCHIRRAIIDKNARIGDNVKIINVDNVQEAARET
DGYFIKSGIVTVIKDALLPSGTVI

pLS (potato AGPase large subunit)

MASVITTENDTQTVFVDMPRLERRRANPKDVAAVILGGGEGTKLFPLTSRTATPAVPVGGCYRLI
DIPMSNCINSAINKIFVLTQYNSAPLNRHIARTYFGNGVSFGDGFVEVLAATQTPGEAGKKWFQG
TADAVRKFIWVFEDAKNKNIENIVVLSGDHLYRMDYMELVQNHIDRNADITLSCAPAEDSRASD
FGLVKIDSRGRVVQFAEKPKGFDLKAMQVDTTLVGLSPQDAKKSPYIASMGVYVFKTDVLLKLL
KWSYPTSNDFGSEIIPAAIDDYNVQAYIFKDYWEDIGTIKSFYNASLALTQEFPEFQFYDPKTPFYT
SPRFLPPTKIDNCKIKDAIISHGCFLRDCSVEHSIVGERSRLDCGVELKDTFMMGADYYQTESEIAS
LLAEGKVPIGIGENTKIRKCIIDKNAKIGKNVSIINKDGVQEADRPEEGFYIRSGIIIILEKATIRDGT
VI

pSS (potato AGPase small subunit)

MAVSDSQNSQTCLDPDASRSVLGIILGGGAGTRLYPLTKKRAKPAVPLGANYRLIDIPVSNCLNS
NISKIYVLTQFNSASLNRHLSRAYASNMGGYKNEGFVEVLAAQQSPENPDWFQGTADAVRQYL
WLFEEHTVLEYLILAGDHLYRMDYEKFIQAHRETDADITVAALPMDEKRATAFGLMKIDEEGRII
EFAEKPQGEQLQAMKVDTTILGLDDKRAKEMPFIASMGIYVISKDVMLNLLRDKFPGANDFGSE
VIPGATSLGMRVQAYLYDGYWEDIGTIEAFYNANLGITKKPVPDFSFYDRSAPIYTQPRYLPPSK
MLDADVTDSVIGEGCVIKNCKIHHSVVGLRSCISEGAIIEDSLLMGADYYETDADRKLLAAKGSV
PIGIGKNCHIKRAIIDKNARIGDNVKIINKDNVQEAARETDGYFIKSGIVTVIKDALIPSGIII

pLS:L2 (potato LS fused to rice L2)

MASVITTENDTQTVFVDMPRLERRRANPKDVAAVILGGGEGTKLFPLTSRTATPAVPVGGCYRLI
DIPMSNCINSAINKIFVLTQYNSAPLNRHIARTYFGNGVSFGDGFVEVLAATQTPGEAGKKWFQG
TADAVRKFIWVFEDAKNKNIENIVVLSGDHLYRMDYMELVQNHIDRNADITLSCAPAEDSRASD
FGLVKIDSRGRVVQFAEKPKGFDLKAMQVDTTLVGLSPQDAKKSPYIASMGVYVFKTDVLLKLL
KWSYPTSNDFGSEIIPAAIDDYNVQAYIFKDYWEDIGTIKSFFDANLALTEQPPKFEFYDPKTPFFT
SPRYLPPARLEKCKIKDAIISDGCSFSECTIEHSVIGISSRVSIGCELKDTMMMGADQYETEEETSK
LLFEGKVPIGIGENTKIRNCIIDMNARIGRNVIIANTQGVQESDHPEEGYYIRSGIVVILKNATIKDG
TVI

L2:pLS (rice L2 fused to potato LS)

MQFMMPLDTNACAQPMRRAGEGAGTERLMERLNIGGMTQEKALRKRCFGDGVTGTARCVFTS
DADRDTPHLRTQSSRKNYADASHVSAVILGGGTGVQLFPLTSTRATPAVPVGGCYRLIDIPMSNC
FNSGINKIFVMTQFNSASLNRHIHHTYLGGGINFTDGSVQVLAATQMPDEPAGWFQGTADAIRKF
MWILEDHYNQNNIEHVVILCGDQLYRMNYMELVQKHVDDNADITISCAPIDGSRASDYGLVKF
DDSGRVIQFLEKPEGADLESMKVDTSFLSYAIDDKQKYPYIASMGIYVLKKDVLLDILKSKYAHL
QDFGSEILPRAVLEHNVKACVFTEYWEDIGTIKSFYNASLALTQEFPEFQFYDPKTPFYTSPRFLPP
TKIDNCKIKDAIISHGCFLRDCSVEHSIVGERSRLDCGVELKDTFMMGADYYQTESEIASLLAEGK
VPIGIGENTKIRKCIIDKNAKIGKNVSIINKDGVQEADRPEEGFYIRSGIIIILEKATIRDGTVI

L2:pLS[K471N] (rice LS fused to potato LS containing a K471N substitution)

MQFMMPLDTNACAQPMRRAGEGAGTERLMERLNIGGMTQEKALRKRCFGDGVTGTARCVFTS
DADRDTPHLRTQSSRKNYADASHVSAVILGGGTGVQLFPLTSTRATPAVPVGGCYRLIDIPMSNC
FNSGINKIFVMTQFNSASLNRHIHHTYLGGGINFTDGSVQVLAATQMPDEPAGWFQGTADAIRKF
MWILEDHYNQNNIEHVVILCGDQLYRMNYMELVQKHVDDNADITISCAPIDGSRASDYGLVKF
DDSGRVIQFLEKPEGADLESMKVDTSFLSYAIDDKQKYPYIASMGIYVLKKDVLLDILKSKYAHL
QDFGSEILPRAVLEHNVKACVFTEYWEDIGTIKSFYNASLALTQEFPEFQFYDPKTPFYTSPRFLPP
TKIDNCKIKDAIISHGCFLRDCSVEHSIVGERSRLDCGVELKDTFMMGADYYQTESEIASLLAEGK
VPIGIGENTKIRKCIIDNNAKIGKNVSIINKDGVQEADRPEEGFYIRSGIIIILEKATIRDGTVI

S2b^{QTC} (rice AGPase small subunit containing QTC replacement)

MNVLASKIFPSRSNVASEQQQSKREKATIDDAKNSSKQTCLDRSVDESVLGIILGGGAGTRLYPL
TKKRAKPAVPLGANYRLIDIPVSNCLNSNISKIYVLTQFNSASLNRHLSRAYGNNIGGYKNEGFVE
VLAAQQSPDNPNWFQGTADAVRQYLWLFEEHNVMEFLILAGDHLYRMDYEKFIQAHRETDSDI
TVAALPMDEKRATAFGLMKIDEEGRIVEFAEKPKGEQLKAMMVDTTILGLDDVRAKEMPYIAS
MGIYVISKNVMLQLLREQFPGANDFGSEVIPGATNIGMRVQAYLYDGYWEDIGTIEAFYNANLGI
TKKPVPDFSFYDRSAPIYTQPRHLPPSKVLDADVTDSVIGEGCVIKNCKIHHSVVGLRSCISEGAII
EDSLLMGADYYETEADKKLLGEKGGIPIGIGKNCHIRRAIIDKNARIGDNVKIINVDNVQEAARET
DGYFIKSGIVTVIKDALLPSGTVI

S2b$^{QTC+L379F}$ (rice AGPase small subunit containing QTC replacement and L379F mutation)

MNVLASKIFPSRSNVASEQQQSKREKATIDDAKNSSKQTCLDRSVDESVLGIILGGGAGTRLYPL
TKKRAKPAVPLGANYRLIDIPVSNCLNSNISKIYVLTQFNSASLNRHLSRAYGNNIGGYKNEGFVE
VLAAQQSPDNPNWFQGTADAVRQYLWLFEEHNVMEFLILAGDHLYRMDYEKFIQAHRETDSDI
TVAALPMDEKRATAFGLMKIDEEGRIVEFAEKPKGEQLKAMMVDTTILGLDDVRAKEMPYIAS
MGIYVISKNVMLQLLREQFPGANDFGSEVIPGATNIGMRVQAYLYDGYWEDIGTIEAFYNANLGI
TKKPVPDFSFYDRSAPIYTQPRHLPPSKVLDADVTDSVIGEGCVIKNCKIHHSVVGFRSCISEGAIIE
DSLLMGADYYETEADKKLLGEKGGIPIGIGKNCHIRRAIIDKNARIGDNVKIINVDNVQEAARETD
GYFIKSGIVTVIKDALLPSGTVI

FIGURE S1. Protein sequences of rice and potato AGPase large subunits (LS) and small subunit (SS) and their fusion proteins used in this study. AGPase subunits from rice endosperm are highlighted in yellow color while those from potato tuber in cyan color. The K471N substitution is highlighted in red color, the QTC replacement in green color, and the L379F mutation in magenta color.

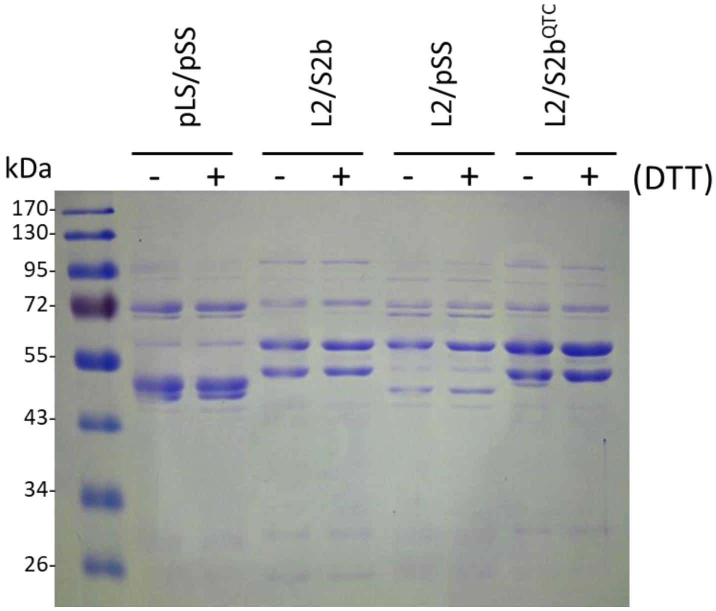

FIGURE S2. SDS-PAGE analysis of dimer formation of AGPase variants. Partially purified enzymes using DEAE-Sepharose FF and TALON-immobilized metal affinity chromatography columns were boiled for 5 min in a SDS sample buffer containing 62.5 mM Tris-HCl (pH 6.8), 2% w/w SDS, 7.5% v/v glycerol in the absence (–) or presence (+) of 10 mM DTT. 2 μg of proteins were applied to SDS-PAGE and immunoblot analysis.

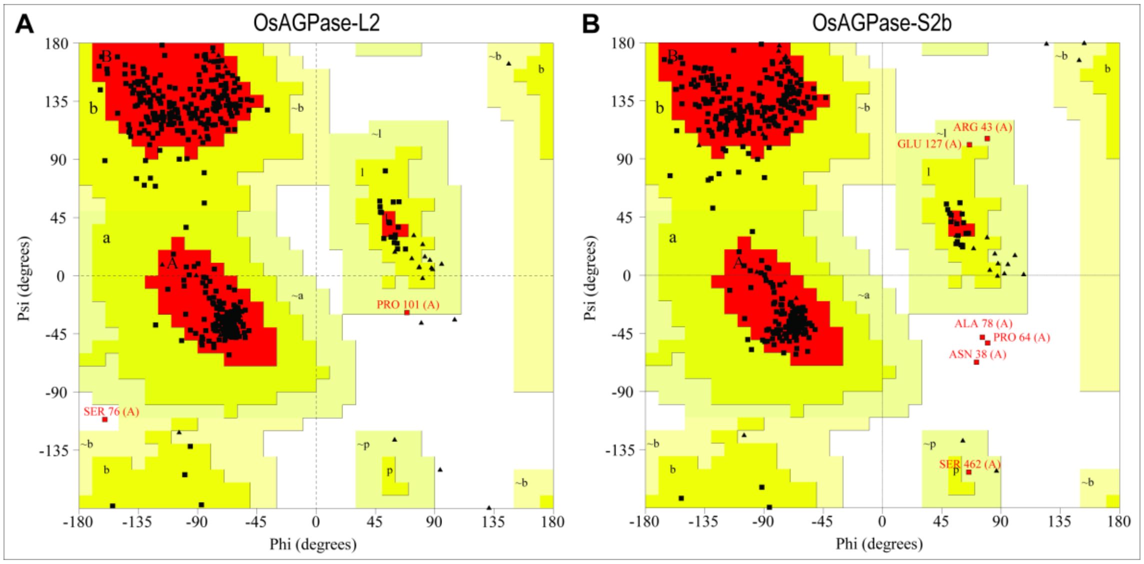

FIGURE S3. Ramachandran plot analysis of AGPase L2 (A) and S2b subunits (B).
